# Evaluation of sST2 Levels in Infants of Mothers with Gestational Diabetes

**DOI:** 10.3390/diagnostics16070982

**Published:** 2026-03-25

**Authors:** Ece Koyuncu, Yücel Pekal, Esin Avcı, Hande Şenol, Musa Turgut, Gülay Sönmez Demir, Özmert M. A. Özdemir

**Affiliations:** 1 Department of Neonatology, Pamukkale University, 20160 Denizli, Turkey; 2 Department of Biochemistry, Pamukkale University, 20160 Denizli, Turkey; 3 Department of Biostatistics, Pamukkale University, 20160 Denizli, Turkey

**Keywords:** gestational diabetes, infant of diabetic mother, sST2, inflammation, newborn

## Abstract

**Objectives:** Gestational diabetes is linked to increased inflammatory and metabolic stress during the neonatal period. Among the biomarkers elucidating the relationship between diabetes and inflammation, the interleukin-33 (IL-33)/ST2 signaling pathway is of particular interest. Research on the IL-33/sST2 axis in pregnancies complicated by diabetes indicates that these biomarkers are associated with maternal metabolic disorders and inflammation. Therefore, evaluating sST2 levels in infants of diabetic mothers is essential for identifying a biological marker of systemic inflammation resulting from intrauterine hyperglycemia and for clarifying the specific risks associated with this condition. The objective of this study was to examine sST2 levels in infants born to diabetic mothers and to assess their association with perinatal inflammation, metabolic stress, and clinical outcomes. **Methods:** This prospective observational study included term infants born at Pamukkale University Medical Faculty Hospital. The study group comprised term infants whose mothers had gestational diabetes, while the control group consisted of term infants born to healthy mothers without diabetes. sST2 levels were measured from serum samples obtained from cord blood at birth using the ELISA method. Factors influencing sST2 levels were analyzed using regression analyses. **Results:** sST2 levels were significantly higher in the diabetic group than in the control group (*p* < 0.001). The incidences of large for gestational age (LGA), small for gestational age (SGA), hypoglycemia, postnatal respiratory distress, and both the frequency and duration of neonatal intensive care unit admissions were also significantly elevated in the diabetic group. Multivariate analysis identified gestational diabetes as independent predictor. **Conclusions:** This study is among the first to demonstrate increased sST2 levels at birth in infants of diabetic mothers. The results indicate that intrauterine exposure to hyperglycemia due to gestational diabetes may be associated with heightened inflammation and metabolic stress in the neonatal period, and that sST2 may serve as a potential biomarker reflecting fetal exposure.

## 1. Introduction

Gestational diabetes (GDM) and pregestational diabetes significantly impact the metabolic, cardiovascular, and respiratory adaptation of newborns. The term infant of a diabetic mother (IDM) refers to infants at increased risk for complications such as macrosomia, neonatal hypoglycemia, respiratory distress, cardiomyopathy, congenital malformations, and persistent pulmonary hypertension. These neonatal morbidities are attributed to fetal hyperinsulinism, oxidative stress, and heightened inflammatory responses resulting from maternal hyperglycemia [1].

Recent research has highlighted the interleukin-33 (IL-33)/ST2 signaling pathway as a key biomarker linking diabetes and inflammation. The soluble form of the ST2 protein (sST2) functions as a “trap receptor” that inhibits the IL-33 receptor, increases in response to inflammatory stimuli, and modulates both innate and adaptive immune responses [2]. Elevated sST2 levels have demonstrated diagnostic and prognostic value in conditions such as sepsis, pulmonary hypertension, myocardial stress, and viral infections. For instance, IL-33 and sST2 concentrations are significantly elevated in neonatal cytomegalovirus infection and are associated with markers of liver damage [3]. Similarly, studies on neonatal sepsis have found strong correlations between sST2 levels and disease severity, supporting its use as a diagnostic marker [4,5]. Evidence for the involvement of sST2 in perinatal cardiopulmonary diseases is also increasing. In infants with pulmonary hypertension, sST2 levels are significantly elevated and correlate with right ventricular failure, indicating its potential as a marker of cardiac stress [6]. Additionally, early increases in sST2 among preterm infants at risk for bronchopulmonary dysplasia have been associated with disease development [7]. Research on the IL-33/sST2 axis in pregnancies complicated by diabetes indicates that these biomarkers are linked to maternal metabolic disorders and inflammation. Elevated IL-33 and sST2 levels in pregnant women with gestational diabetes correlate positively with insulin resistance markers, suggesting a role for the IL-33/sST2 system in hyperglycemia-induced inflammatory processes [8].

Increasing evidence links the IL-33/ST2 system to metabolic and inflammatory pathways, suggesting that circulating sST2 could serve as a valuable biomarker for these processes. Evaluating sST2 levels in infants of diabetic mothers may clarify the relationship between intrauterine hyperglycemia, inflammation, and subsequent cardiometabolic risk. To date, no studies have systematically examined neonatal sST2 levels in this population. The primary objective of this study was to measure serum sST2 levels in infants born to diabetic mothers and to assess their association with neonatal clinical outcomes and metabolic stress.

## 2. Materials and Methods

The study was conducted on term infants born at Pamukkale University Medical Faculty Hospital. Patients were divided into two groups: the study group and the control group. The study group included term infants whose mothers had gestational diabetes. Patients with a gestational age of less than 37 weeks, sepsis or congenital infection, maternal chorioamnionitis, history of fever infection, or lack of family consent were excluded. The control group comprised healthy term newborns delivered during the same period and at the same institution as the study group. Inclusion criteria specified that mothers must not have diabetes and that informed consent was obtained. Although formal matching was not conducted, maternal and neonatal baseline characteristics, such as maternal age and parity, were recorded and compared between groups. Maternal body mass index (BMI) data were unavailable for all participants; therefore, BMI-based matching or analysis was not feasible. Maternal obesity status, defined by BMI thresholds, was instead recorded and analyzed as a categorical proxy variable. Participants with a prior diagnosis of diabetes or overt diabetes during pregnancy were excluded from the study. Gestational diabetes mellitus was identified using a 75 g oral glucose tolerance test in accordance with the 2013 World Health Organization criteria [9].

A priori sample size calculation was conducted using G*Power software (version 3.1.9.7). The analysis utilized a two-tailed independent samples *t*-test with a significance level of α = 0.05 and 80% statistical power. While the reference study reported a large effect size (d = 1.882), a more conservative effect size (d = 0.8) was selected to mitigate the risk of overestimation [4]. Based on these parameters, the minimum required sample size was determined to be 52 participants (26 per group). Ultimately, 56 participants were included in the study, comprising 29 individuals in the diabetic group and 27 in the control group. Ethical committee approval was obtained from the Pamukkale University Faculty of Medicine Non-Interventional Clinical Research Ethics Committee on 14 June 2023 with the number E-60116787-020-380655.

After obtaining ethical committee approval, at least 2 mL of cord blood was collected from the infants at birth, and serum samples were obtained from the clotted blood by centrifuging at 3500 rpm for 10 min, then incubating for at least 25 min. The serum separated into Eppendorf tubes was stored at −20 degrees Celsius or lower until the day of analysis. sST2 analyses were performed on serum brought to room temperature on the day of the study.

Patients’ demographic data, complications that may have developed due to gestational diabetes, hospitalizations, and hospitalization durations were recorded.

Hypoglycemia diagnosis in patients was evaluated according to the criteria of the American Academy of Pediatrics. If the infant was symptomatic, blood glucose levels below 40 mg/dL were considered hypoglycemia; if asymptomatic, blood glucose levels below 25 mg/dL in the first 4 h postnatally and below 35 mg/dL between 4 and 24 h postnatally were considered hypoglycemia. After 24 h postnatally, blood glucose levels below 45 mg/dL were considered hypoglycemia [10]. In term newborns, total calcium <8 mg/dL was considered hypocalcemia, and serum magnesium levels below 1.6 mg/dL were considered hypomagnesemia [11]. A venous hematocrit value above 65% was considered polycythemia [12]. The level of jaundice requiring treatment was assessed according to the recommendations of the Turkish Neonatology Association’s 2023 Update of the Approach, Monitoring, and Treatment Guide for Neonatal Jaundice [13]. Respiratory distress was defined as the presence of clinical signs such as tachypnea, nasal flaring, grunting, or intercostal retractions. Transient tachypnea of the newborn (TTN) was diagnosed based on early onset respiratory distress with characteristic clinical and radiological findings and resolution within the first days of life [14]. Respiratory distress syndrome (RDS) was defined based on clinical signs of respiratory distress accompanied by typical radiographic findings and the need for respiratory support, including surfactant therapy [14].

sST2 levels were measured using enzyme-linked immunosorbent assay (ELISA) kits from Sunlong Quick Step Human Soluble suppression tumorigenicity (Sunlong Biotech Co. Ltd., Hangzhou, China). The ELISA kit uses a sandwich ELISA method. Tests were performed at 25 °C on the day of analysis. Absorbance was measured at 450 nm using a BioTek ELISA reader (BioTek Instruments, Inc., Winooski, VT, USA), and concentrations were determined using Gen5 software version 3.03. The intra-assay coefficient of variation (CV) levels for sST-2 were <10%, while the inter-assay CV was <12%. All samples were analyzed in duplicate to minimize analytical variability. The sST-2 reading range was 6–220 pg/mL, and the kit sensitivity was 1 pg/mL.

### Statistical Analyses

All statistical analyses were performed using SPSS 25.0 (IBM SPSS Statistics 25 software (IBM Corp., Armonk, NY, USA)). Continuous variables were reported as mean ± standard deviation and median (25th–75th percentiles). The Shapiro–Wilk test was used to determine whether the data were normally distributed. The independent-samples *t*-test was used to compare independent groups when the parametric test assumptions were met. The Mann–Whitney U test was used to compare independent groups when the assumptions of parametric tests were not met. Differences between categorical variables were analyzed using the Chi-square test and Fisher’s exact test. To investigate the effects of independent factors on sST2 levels, we used univariate and multivariate linear regression models. *p* < 0.05 was considered statistically significant.

## 3. Results

A total of 56 patients were included in the study, comprising 29 mothers with gestational diabetes and 27 control group participants. The demographic data of the patients included in the study are presented in Table 1. No statistically significant differences were observed between the groups regarding maternal age, parity, gestational week, birth weight, birth length, head circumference, or mode of delivery. These findings indicate that the groups were comparable at baseline.

Data on maternal diseases, medications used during pregnancy, and smoking are presented in Table 2. No statistically significant differences were found between groups in terms of medicines used by mothers and smoking. Additionally, maternal obesity status, used as a categorical proxy for maternal BMI, did not differ significantly between the groups. In the control group, maternal hypothyroidism and L-thyroxine (Lt4) use were significantly higher than in the diabetic group.

The neonatal complications observed in patients with gestational diabetes are shown in Table 3. When the weight variable was examined by week, the rates of large for gestational age (LGA) and small for gestational age (SGA) in the diabetic group were found to be statistically significantly higher than in the control group. In the diabetic group, hypoglycemia, postnatal respiratory distress, frequency of admission to the neonatal intensive care unit, and length of stay were found to be significantly higher than in the control group.

**Figure 1:**
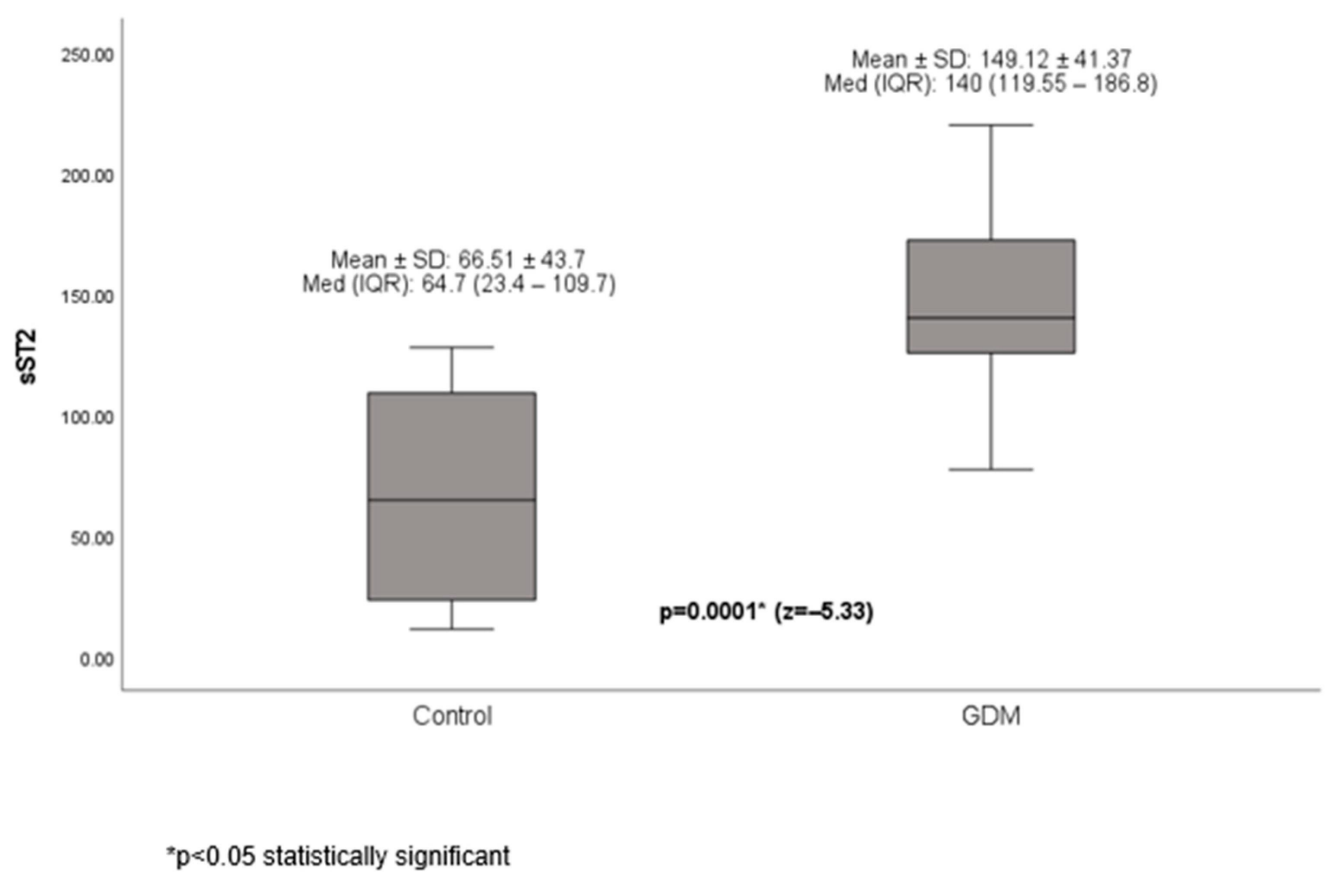
sST2 levels in the diabetic group and the control group.

sST2 levels were significantly higher in the diabetic group than in the control group (Figure 1).

In the analysis of factors affecting sST2 levels using univariate linear regression models, the presence of gestational diabetes in the mother was found to have a statistically significant effect on the increase in sST2 levels. In addition, maternal multivitamin use, the presence of hypoglycemia in the infant, and admission to the neonatal intensive care unit were also found to have a significantly elevating effect on sST2 levels. Increased gestational age, increased head circumference at birth, maternal use of Lt4, maternal comorbidities, and the presence of hypothyroidism were also found to have a significant downward effect on sST2 levels (Table 4).

In the multivariate model, including variables with significant effects in univariate analyses, only GDM was independently associated with sST2 levels. The presence of GDM was found to have a statistically significant, increasing effect on sST2 levels (Table 4).

## 4. Discussion

The significantly elevated sST2 levels observed in infants of diabetic mothers in this study suggest that this may be a biochemical indicator of increased inflammatory or metabolic stress response in the fetus exposed to intrauterine hyperglycemia. It is known that sST2 regulates the immune response as a soluble receptor for IL-33 [2]. It has previously been shown that oxidative stress and immune activation in diabetic pregnancies may affect the IL-33/ST2 axis [15], and our study contributes significantly to the literature by being one of the few to evaluate this biological effect at the level of the newborn. In this regard, our study presents a unique approach that provides neonatal data on the possible transfer of maternal biological processes to the fetus.

Studies have reported elevated sST2 levels in pregnant women with gestational diabetes and their association with metabolic parameters [8]. However, data on how these changes translate into a biochemical profile in the fetus are minimal. Our study presents findings that constitute early evidence that a significant elevation in sST2 measured at birth in IDM may reflect maternal diabetes-related inflammatory/metabolic processes into the neonatal period. This result complements the maternal biomarker literature and suggests that sST2 may be a potential marker reflecting the degree of neonatal involvement in the context of diabetic pregnancy. Conventional inflammatory markers, including CRP and IL-6, were not assessed in this study as the primary objective was to examine sST2 as a pathway-specific biomarker.

While the neonatal literature provides evidence linking sST2 to inflammatory responses and disease severity, data specific to infants of diabetic mothers remain limited. Studies on sepsis and viral infections have demonstrated associations between sST2 and inflammation [4,5]. Elevated sST2 levels have also been linked to poor prognosis in cardiopulmonary conditions such as pulmonary hypertension and bronchopulmonary dysplasia [6,7]. These findings suggest that the increased sST2 levels observed in this study may indicate stress or an inflammatory burden during the neonatal period, providing a foundation for future research on potential neonatal biological effects.

Experimental and clinical studies have shown that the IL-33/ST2 axis is associated with cardiac stress and tissue remodeling processes, in addition to inflammation. In respiratory syncytial virus (RSV)-associated pulmonary hypertension models, this pathway has been reported to increase the fibrotic response, and pathological processes have been reported to decrease in the absence of sST2 [16]. High sST2 levels have also been reported to be associated with mortality and readmission risk in populations undergoing pediatric congenital heart surgery [17]. Substantial evidence indicates that sST2 is associated with clinical outcomes such as cardiac fibrosis, heart failure, and mortality in adult populations [18]. In this study, the incidence of cardiovascular pathology was low, with only two cases of patent ductus arteriosus (PDA), one atrial septal defect (ASD), and one case of septal hypertrophy identified. Consequently, the relationship between sST2 levels and cardiac structure or function could not be comprehensively evaluated. The limited number of cases resulted in insufficient statistical power to assess potential associations between sST2 levels and these rare cardiovascular outcomes. Furthermore, the absence of follow-up data prevents conclusions regarding the role of sST2 in predicting long-term cardiometabolic outcomes. Thus, sST2 should be considered a potential biomarker reflecting current inflammatory and metabolic status rather than a predictor of future risk. Future studies with larger sample sizes and longitudinal follow-up are necessary to investigate this relationship.

Although elevated sST2 levels in the diabetic group were associated with neonatal morbidities such as hypoglycemia, respiratory distress, and neonatal intensive care unit admission in univariate analyses, these associations did not remain significant after adjustment in the multivariable model and were not identified as independent predictors. This attenuation may be partly attributed to the relatively low incidence of these morbidities within the cohort and the complex interrelationships among neonatal clinical variables. Conditions such as hypoglycemia, respiratory distress, and NICU admission are closely linked to the overall clinical status of the newborn and are often influenced by factors such as gestational age, metabolic adaptation, and perinatal stress. These variables may function as mediators or confounders in their association with sST2 levels. Consequently, rather than serving as independent determinants, these clinical conditions may reflect the broader physiological stress and inflammatory response of the neonate, which subsequently influences sST2 levels. Maternal vitamin use was associated with sST2 levels in univariate analysis; however, this association did not persist in the multivariable model, indicating that it is not an independent predictor. In this cohort, vitamin supplementation comprised a prenatal preparation containing folic acid, vitamin D, vitamin B12, iodine, and zinc. Although these micronutrients are recognized for their roles in immune regulation and oxidative stress pathways, the observed association may be attributable to confounding factors rather than a direct biological effect [19]. Consequently, this finding should be interpreted with caution. In the multivariate model, only gestational diabetes remained an independent predictor, suggesting that the rise in sST2 may be primarily related to the intrauterine metabolic environment.

In our study, hypothyroidism and L-thyroxine use were more prevalent among mothers in the control group, potentially affecting baseline comparability and introducing a possible source of selection bias. Although thyroid disorders can influence inflammatory pathways, these variables were included in the multivariable analysis and did not show an independent association with sST2 levels. Consequently, although a confounding effect cannot be entirely excluded, the observed difference is unlikely to have significantly influenced the main findings.

This study’s strengths include its novel contribution to the limited literature on sST2 levels at birth in IDM, the comprehensive assessment of biomarker levels alongside a wide range of clinical variables, and the robust evaluation of independent predictors through multivariate modeling. One limitation of this study is the relatively small sample size, which may limit statistical power and the generalizability of the findings. The limited sample size also restricts the ability to detect associations with less frequent clinical outcomes and increases the risk of residual confounding. Another limitation is the absence of IL-33 level measurements, which precluded a comprehensive evaluation of the IL-33/ST2 signaling pathway. Future research incorporating both IL-33 and sST2 measurements may provide additional mechanistic insights. Additionally, this study relied on the 2011 American Academy of Pediatrics (AAP) criteria to define neonatal hypoglycemia. While more recent guidelines and expert recommendations advocate for higher glucose threshold values, the 2011 criteria are still routinely used in our unit’s clinical practice because they take into account transitional physiology and aim to minimize unnecessary interventions during the early postnatal period. The use of different diagnostic thresholds may influence the identification and management of hypoglycemia and may affect comparability with studies using updated criteria. Maternal BMI was recorded as a categorical variable for obesity status rather than as a continuous measure. Additionally, data on the previous history of gestational diabetes were unavailable. These methodological limitations may have resulted in residual confounding. As this is a single-center study, our cohort may not be fully representative of broader neonatal populations. While the uniform clinical setting likely reduced inter-center variability, the possibility of selection bias and residual confounding related to study design remains. Future prospective, longitudinal studies with larger cohorts are needed to elucidate changes in the IL-33/sST2 axis over time, its relationship with the diabetic intrauterine environment, and its impact on both short- and long-term neonatal outcomes.

## 5. Conclusions

The present study found that sst2 levels were significantly higher in term newborns born to mothers with gestational diabetes compared to the control group. These results indicate that sst2 may serve as a potential biomarker reflecting fetal effects in diabetic pregnancies. Further research with larger sample sizes and extended follow-up is required to assess maternal factors influencing this mechanism, the clinical significance of sst2, and its association with complications.

## Figures and Tables

**Table 1:** The demographic data of the patients.

		Control	Diabetic	*p*
Gender	Female	13 (48.1%)	18 (62.1%)	0.295 (cs = 1.096)
	Male	14 (51.9%)	11 (37.9%)	0.295 (cs = 1.096)
gestational week	Mean ± SD	38.19 ± 1.04	37.83 ± 0.97	0.129 (z = −1.52)
	Med (IQR)	38 (38–38)	38 (37–38)	0.129 (z = −1.52)
birth weight	Mean ± SD	3212.96 ± 329.71	3332.76 ± 612.51	0.363 (t = −0.920)
	Median (IQR)	3240 (2990–3415)	3265 (2932.5–3725)	0.363 (t = −0.920)
birth length	Mean ± SD	48.8 ± 2.35	49.07 ± 2.1	0.648 (t = −0.459)
	Median (IQR)	49 (47.5–51)	49.5 (47.75–51)	0.648 (t = −0.459)
birth head circumference	Mean ± SD	35.02 ± 1.05	34.19 ± 1.67	0.113 (z = −1.584)
	Median (IQR)	35 (34.5–36)	35 (33–35.25)	0.113 (z = −1.584)
mother’s age	Mean ± SD	29.15 ± 5.01	30.03 ± 5.01	0.511 (t = −0.661)
	Median (IQR)	30 (25–33)	29 (27–33.5)	0.511 (t = −0.661)
Number of pregnancies	Mean ± SD	2.63 ± 1.57	2.34 ± 1.4	0.531 (z = −0.626)
	Median (IQR)	2 (1–3)	2 (1–3)	0.531 (z = −0.626)
Mode of delivery	nsvd	3 (11.1%)	2 (6.9%)	0.664 γ
	c/s	24 (88.9%)	27 (93.1%)	0.664 γ
multiple pregnancy	none	27 (100%)	29 (100%)	-

SD: Standard deviation; Med (IQR): Median (25th–75th percentiles); t: independent samples *t* test; z: Mann–Whitney U test; cs: Chi-square test; γ: Fisher exact test.

**Table 2:** Maternal diseases and medications used during pregnancy.

		Control	Diabetic	*p*
mother medication	Present	25 (92.6%)	29 (100%)	0.228 γ
iron	Present	24 (88.9%)	29 (100%)	0.106 γ
vitamin	Present	20 (74.1%)	27 (93.1%)	0.073 γ
inhaled steroid	Present	2 (7.4%)	1 (3.4%)	0.605 γ
Lt4	Present	6 (22.2%)	1 (3.4%)	0.048 * γ
antihypertensive	Present	0 (0%)	1 (3.4%)	1 γ
ursactive	Present	0 (0%)	1 (3.4%)	1 γ
n-acetyl cystein	Present	0 (0%)	1 (3.4%)	1 γ
sodium alginate	Present	0 (0%)	1 (3.4%)	1 γ
sertraline	Present	1 (3.7%)	0 (0%)	0.482 γ
omega 3 fatty acid	Present	1 (3.7%)	0 (0%)	0.482 γ
maternal disease	Present	15 (55.6%)	6 (20.7%)	0.007 * (cs = 7.252)
asthma	Present	3 (11.1%)	1 (3.4%)	0.343 γ
hypothyroidism	Present	7 (25.9%)	1 (3.4%)	0.023 * γ
hypertension	Present	0 (0%)	1 (3.4%)	1 γ
pregnancy cholestasis	Present	0 (0%)	1 (3.4%)	1 γ
achalasia	Present	0 (0%)	1 (3.4%)	1 γ
obesity	Present	4 (14.8%)	1 (3.4%)	0.185 γ
arrhythmia	Present	1 (3.7%)	0 (0%)	0.482 γ
coagulation disorder	Present	1 (3.7%)	0 (0%)	0.482 γ
COPD	Present	1 (3.7%)	0 (0%)	0.482 γ
thalassemia trait	Present	1 (3.7%)	0 (0%)	0.482 γ
maternal smoking	Present	3 (11.1%)	1 (3.4%)	0.343 γ
blood sugar regulation	diet	-	19 (65.5%)	-
blood sugar regulation	insulin	-	10 (34.5%)	-

* *p* < 0.05 statistically significant; cs: Chi-square test; γ: Fisher exact test. COPD: chronic obstructive pulmonary disease, Lt4: L-thyroxine.

**Table 3:** The neonatal complications of gestational diabetes.

		Control	Diabetic	*p*
Birth weight	AGA	27 (100%)	22 (75.9%)	0.006 * (cs = 10.144)
	LGA	0 (0%)	5 (17.2%)	0.006 * (cs = 10.144)
	SGA	0 (0%)	2 (6.9%)	0.006 * (cs = 10.144)
hypoglycemia		0 (0%)	5 (17.2%)	0.05 * γ
hypoglycemia treatment	nutrition		3 (60%)	-
	IV glucose		2 (40%)	-
respiratory distress		0 (0%)	6 (20.7%)	0.024 * γ
cardiac anomaly		0 (0%)	4 (13.8%)	0.112 γ
	PDA	-	2 (50%)	-
	ASD	-	1 (25%)	-
	septal hypertrophy	-	1 (25%)	-
jaundice requiring treatment		1 (3.7%)	5 (17.2%)	0.195 γ
hemoglobin	Mean ± SD	-	18.34 ± 2.17	-
	Median (IQR)	-	18.3 (16.5–20.25)	-
hematocrit	Mean ± SD	-	54.74 ± 6.88	-
	Median (IQR)	-	53.9 (48.1–62.1)	-
calcium	Mean ± SD	-	9.24 ± 0.53	-
	Median (IQR)	-	9.3 (8.95–9.5)	-
magnesium	Mean ± SD	-	1.91 ± 0.18	-
	Median (IQR)	-	1.9 (1.8–1.95)	-
NICU admission		3 (11.1%)	14 (48.3%)	0.003 * (cs = 9.135)
Reason for admission	TTN	1 (33.3%)	6 (42.6%)	-
	ear injury	0	1 (7.1%)	-
	jaundice	1 (33.3%)	2 (14.4%)	-
	RDS	0	1 (7.1%)	-
	hypoglycemia	0	2 (14.4%)	-
	Weight loss	1 (33.3%)	2 (14.4%)	-
Hospitalization duration	Mean ± SD	0.86 ± 3.07	2.83 ± 5.15	0.015 * (z = −2.434)
	Median (IQR)	0 (0–0)	0 (0–4)	0.015 * (z = −2.434)

* *p* < 0.05 statistically significant; SD: Standard deviation; Median (IQR): Median (25th–75th percentiles); z: Mann–Whitney U test; cs: Chi-square test; γ: Fisher exact test. AGA: appropriate for gestational age; LGA: Large for gestational age; SGA: Small for gestational age; PDA: patent ductus arteriosus; ASD: Atrial septal defect; NICU: neonatal intensive care unit; TTN: transient tachypnea of newborn; RDS: respiratory distress syndrome.

**Table 4:** Factors affecting sST2 levels.

Univariate Models	Std. Beta	t	*p*	%95 C.I. Lower–Upper
Group (GDM; ref: Control)	0.703	7.266	0.0001 *	59.814–105.398
gestational week	−0.301	−2.316	0.024 *	−32.924–−2.373
Gender (Male; ref: Female)	−0.04	−0.294	0.77	−36.913–27.472
birth weight	−0.059	−0.433	0.667	−0.04–0.026
birth length	−0.105	−0.774	0.442	−10.091–4.471
Birth head circumference	−0.343	−2.687	0.01 *	−24.4–−3.546
Weekly weight last	0.234	1.767	0.083	−5.589–88.585
Birth method (CS; ref: Nsvy)	0.083	0.614	0.542	−38.832–73.112
mother’s age	−0.051	−0.375	0.709	−3.842–2.632
number of pregnancies	−0.217	−1.631	0.109	−19.373–1.992
5-min Apgar	−0.004	−0.03	0.976	−31.049–30.12
mother medication (Present; ref: Absent)	0.181	1.353	0.182	−27.594–142.164
iron (Present; ref: Absent)	0.207	1.557	0.125	−15.533–123.636
vitamin (Present; ref: Absent)	0.274	2.096	0.041 *	1.896–85.772
Inhaled steroid (Present; ref: Absent)	0.001	0.008	0.993	−70.837–71.423
Lt4 (Present; ref: Absent)	−0.281	−2.154	0.036 *	−96.404–−3.457
antihypertensive (Present; ref: Absent)	0.039	0.287	0.775	−103.528–138.168
ursactive (Present; ref: Absent)	0.043	0.316	0.753	−101.777–139.879
N-acetyl cystein (Present; ref: Absent)	0.043	0.316	0.753	−101.777–139.879
sodium alginate (Present; ref: Absent)	0.121	0.898	0.373	−66.276–173.818
sertraline (Present; ref: Absent)	−0.218	−1.641	0.107	−214.647–21.418
omega3 fa (Present; ref: Absent)	−0.225	−1.696	0.096	−217.512–18.174
Maternal disease (Present; ref: Absent)	−0.291	−2.237	0.029 *	−66.969–−3.671
Asthma (Present; ref: Absent)	−0.146	−1.084	0.283	−94.789–28.259
Hypothyroidism (Present; ref: Absent)	−0.265	−2.024	0.048 *	−88.668–−0.412
hypertension (Present; ref: Absent)	0.039	0.287	0.775	−103.528–138.168
pregnancy cholestasis (Present; ref: Absent)	0.043	0.316	0.753	−101.777–139.879
Achalasia (Present; ref: Absent)	0.121	0.898	0.373	−66.276–173.818
obesity (Present; ref: Absent)	−0.244	−1.852	0.069	−104.785–4.141
arrhythmia (Present; ref: Absent)	0.008	0.059	0.953	−117.361–124.511
Coagulation disorder (Present; ref: Absent)	−0.218	−1.641	0.107	−214.647–21.418
COPD (Present; ref: Absent)	−0.13	−0.96	0.341	−177.336–62.507
Thalassemia trait (Present; ref: Absent)	0.038	0.281	0.78	−103.939–137.765
Blood sugar regulation (Diet; ref: Insulin)	−0.079	−0.414	0.682	−20.228–13.44
Maternal smoking (Present; ref: Absent)	−0.089	−0.653	0.516	−82.126–41.765
hypoglycemia (Present; ref: Absent)	0.313	2.418	0.019 *	11.005–117.709
Respiratory distress (Present; ref: Absent)	0.162	1.203	0.234	−20.441–81.766
cardiac anomaly present (Present; ref: Absent)	0.055	0.406	0.686	−49.51–74.68
hemoglobin	0.297	1.613	0.118	−1.534–12.831
hematocrit	0.251	1.345	0.19	−0.792–3.807
calcium	0.084	0.44	0.663	−24.028–37.156
magnesium	−0.003	−0.016	0.987	−92.695–91.27
hyperbilirubinemia	−0.189	−1	0.326	−62.076–21.386
NICU admission (Present; ref: Absent)	0.307	2.367	0.022 *	5.992–72.303
settling time	0.084	0.587	0.56	−2.742–5.005
Multiple Model 1				
Group (GDM; ref: Control)	0.611	5.089	0.0001 *	43.482–100.189
vitamin	0.123	1.181	0.243	−13.744–52.932
hipotiroidi	−0.057	−0.542	0.590	−44.669–25.681
hypoglycemia (Present; ref: Absent)	0.120	1.129	0.264	−19.29–68.77
NICU admission (Present; ref: Absent)	0.014	0.122	0.904	−26.838–30.296

* *p* < 0.05 statistically significant effect; Std. Beta: Standardized Beta Coefficient; 95% C.I: 95% Confidence Interval. COPD: chronic obstructive pulmonary disease, Lt4: L-thyroxine.

## Data Availability

The data presented in this study are available on request from the corresponding author. The data are not publicly available due to ethical and confidentiality restrictions.

## Abbreviations

The following abbreviations are used in this manuscript:

GDM	Gestational diabetes
IDM	Infant of diabetic mother
IL-33	Interleukin-33
sST2	Soluble form of the ST2 protein
WHO	World Health Organization
ELISA	Enzyme-linked immunosorbent assay
COPD	Chronic obstructive pulmonary disease
Lt4	L-thyroxine
AGA	Appropriate for gestational age
LGA	Large for gestational age
SGA	Small for gestational age
PDA	Patent ductus arteriosus
ASD	Atrial septal defect
NICU	Neonatal intensive care unit
TTN	Transient tachypnea of newborn
RDS	Respiratory distress syndrome
RSV	Respiratory syncytial virus
